# Sentinel Surveillance for Congenital Rubella Syndrome — India, 2016–2017

**DOI:** 10.15585/mmwr.mm6736a4

**Published:** 2018-09-14

**Authors:** Manoj Murhekar, Ashish Bavdekar, Asha Benakappa, Sridhar Santhanam, Kuldeep Singh, Sanjay Verma, Gajanan N. Sapkal, Nivedita Gupta, Valsan Philip Verghese, Rajlakshmi Viswanathan, Asha Mary Abraham, Shyama Choudhary, Gururajrao N. Deshpande, Suji George, Garima Goyal, Parul Chawla Gupta, Ishani Jhamb, Deepa John, Swetha Philip, Sandeep Kadam, Ravinder Kaur Sachdeva, Praveen Kumar, Anjali Lepcha, S. Mahantesh, S. Manasa, Urvashi Nehra, Sanjay Kumar Munjal, Vijaya Lakshmi Nag, Sadanand Naik, Naga Raj, Jagat Ram, R.K. Ratho, C.G. Raut, Manoj Kumar Rohit, R. Sabarinathan, Sanjay Shah, Pratibha Singh, Mini P. Singh, Ashish Tiwari, Neelam Vaid

**Affiliations:** ^1^National Institute of Epidemiology, Chennai, India; ^2^KEM Hospital Research Centre, Pune, India; ^3^Indira Gandhi Institute for Child Health, Bengaluru, India; ^4^Christian Medical College, Vellore, India; ^5^All India Institute of Medical Sciences, Jodhpur, India; ^6^Postgraduate Institute of Medical Education and Research, Chandigarh, India; ^7^National Institute of Virology, Pune, India; ^8^Indian Council of Medical Research, New Delhi, India; ^9^National Institute of Virology, Bengaluru Unit, Bengaluru, India.

Rubella infection during pregnancy can result in miscarriage, fetal death, stillbirth, or a constellation of congenital malformations known as congenital rubella syndrome (CRS). The 11 countries in the World Health Organization (WHO) South-East Asia Region are committed to the elimination of measles and control of rubella and CRS by 2020. Until 2016, when the Government of India’s Ministry of Health and Family Welfare and the Indian Council of Medical Research initiated surveillance for CRS in five sentinel sites, India did not conduct systematic surveillance for CRS. During the first 8 months of surveillance, 207 patients with suspected CRS were identified. Based on clinical details and serologic investigations, 72 (34.8%) cases were classified as laboratory-confirmed CRS, four (1.9%) as congenital rubella infection, 11 (5.3%) as clinically compatible cases, and 120 (58.0%) were excluded as noncases. The experience gained during the first phase of surveillance will be useful in expanding the surveillance network, and data from the surveillance network will be used to help monitor progress toward control of rubella and CRS in India.

Rubella is a common cause of childhood febrile rash illness in India, typically associated with mild illness; however, infection during the first trimester of pregnancy can severely affect the fetus, resulting in spontaneous abortion, stillbirth, or CRS (*1*,*2*). In 2010, among an estimated 103,000 infants with CRS born globally, 46% were born in the South-East Asia Region (*3*). The Government of India is committed to eliminating measles and controlling rubella and CRS by 2020. Maintaining high population immunity to rubella, creating a network of laboratories, and developing and sustaining a case-based surveillance system are the principal strategies for elimination of measles and control of rubella and CRS (*3*).

In 2017, India introduced measles-rubella vaccine nationwide and launched a mass vaccination campaign targeting children aged 9 months to 14 years in five states or union territories (*4*), with plans for phased expansion to the remaining states. Outbreak-based and laboratory-supported measles and rubella surveillance was established in the country in 2005 (*5*,*6*). Although several published studies in India have examined the prevalence of CRS among different population groups, including patients with cataracts and other ocular abnormalities, hearing loss, mental retardation, cardiac defects, and other congenital anomalies, there was no systematic CRS surveillance system (*7*). To address this gap, the Indian Council of Medical Research and the Ministry of Health and Family Welfare initiated laboratory-supported surveillance for CRS in five sentinel sites in five Indian states in December 2016.

CRS surveillance is focused on identifying suspected CRS cases among infants aged 0–11 months who are patients in pediatrics; ear, nose, and throat (ENT); ophthalmology; and cardiology outpatient departments of the sentinel hospitals. Suspected CRS cases also are identified during the routine clinical examination of newborn babies born at the sentinel sites.

According to the case definitions adapted from WHO-recommended standards for CRS surveillance (Box) (*3*), all infants with suspected CRS are referred to the site surveillance coordinator (a pediatrician) for a complete physical examination. After obtaining written informed consent from the parents to enroll the infant into the surveillance system, demographic, epidemiologic, and clinical information is obtained, and a 1mL blood specimen is collected from the infant. Among infants aged 6–11 months at the time of enrollment, additional blood specimens are collected one month after the first specimen and measured quantitatively to document a sustained rise in immunoglobulin G (IgG) antibodies against rubella. Serum is tested for immunoglobulin M (IgM) and IgG antibodies against rubella using a commercial enzyme-linked immunosorbent assay. In addition, all infants aged <6 months at the time of enrollment have oropharyngeal swabs collected and transported to the National Institute of Virology in Pune for reverse transcription–polymerase chain reaction (RT-PCR) testing and genotyping conducted according to WHO guidelines (*8*).

BOX Case definitions* for congenital rubella syndrome (CRS) surveillance — Congenital Rubella Syndrome Sentinel Surveillance, India, December 2016–July 2017

**Table:** 

**Suspected CRS.** The presence of any of the following conditions in an infant: Structural heart defect (excluding PDA^†^ or PFO^†^ in infants <37 weeks gestational age). Hearing impairment.^§^ One or more of the following eye signs: cataract, microphthalmos, microcornea, congenital glaucoma, and pigmentary retinopathy. Maternal history of suspected or confirmed rubella infection during pregnancy. Strong clinical suspicion. **Clinically confirmed CRS.** The detection by a physician of two clinical signs from group A or one from group A and one from group B in an infant: **Group A:** Cataract(s), congenital glaucoma, pigmentary retinopathy, congenital heart defect, or hearing loss. **Group B:** Microcephaly, developmental delay, meningoencephalitis, splenomegaly, purpura, radiolucent bone disease, or jaundice with onset within 24 hours after birth. **Laboratory-confirmed CRS.** The presence in an infant of one condition from Group A (above) and one of the following laboratory criteria: Detection of rubella IgM antibody; or Sustained detectable rubella IgG antibody level, as determined on at least two occasions at age 6–12 months, in the absence of receipt of rubella vaccine. **Congenital rubella infection.** Absence of any clinical signs from group A in an infant with a positive rubella-specific IgM test. **Clinically compatible case.** Signs or symptoms of CRS in a patient from whom a blood specimen could not collected. **Excluded noncase.** A negative serologic result for rubella, irrespective of clinical signs present in an infant. **Abbreviations:** IgG = immunoglobulin G; IgM = immunoglobulin M; PDA = patent ductus arteriosus; PFO = patent foramen ovale. *Adapted from World Health Organization. Strategic plan for measles elimination and rubella and congenital rubella syndrome control in the South-East Asia Region. New Delhi, India: World Health Organization Regional Office for South-East Asia; 2015. (http://www.searo.who.int/entity/immunization/documents/sear_mr_strategic_plan_2014_2020.pdf). ^†^ Confirmed by echocardiography. ^§^ Confirmed by auditory brainstem response or auditory steady-state response audiometry.

During December 2016–July 2017, the surveillance system identified 207 patients with suspected CRS (Table 1). Forty-one (19.8%) suspected CRS patients were detected on routine newborn examination; the remaining 166 were identified through pediatrics, ophthalmology, cardiology, or ENT outpatient departments at sentinel sites. Overall, 145 (70%) patients with suspected CRS were aged ≤5 months (median age = 3 months; interquartile range = 0–7 months) at the time of diagnosis; infants with CRS were from 11 states.

**TABLE 1:** Characteristics of suspected cases of congenital rubella syndrome (CRS) (N = 207) — Congenital Rubella Sentinel Surveillance System, India, December 2016–July 2017

Characteristic of patients	No. of cases (%)
**Sentinel site (state)**	
Postgraduate Institute of Medical Education and Research, Chandigarh (Punjab/Haryana)	60 (29.0)
All India Institute of Medical Sciences, Jodhpur (Rajasthan)	49 (23.7)
KEM Hospital, Pune (Maharashtra)	36 (17.4)
Indira Gandhi Institute for Child Health, Bengaluru (Karnataka)	35 (16.9)
Christian Medical College, Vellore (Tamil Nadu)	27 (13.0)
**Department first consulted**	
Pediatrics	85 (41.1)
Neonatology	41 (19.8)
Ophthalmology	44 (21.3)
Cardiology	22 (10.6)
Ear, nose, and throat	7 (3.4)
Other	8 (3.9)
**Criteria for suspecting CRS**	
Structural heart defect	135 (65.2)
Eye signs	94 (45.4)
Maternal history of fever with rash during pregnancy	41 (19.8)
Hearing impairment	37 (17.9)
Clinically suspected	11 (5.3)
**Age at diagnosis**	
<1 month	56 (27.1)
1–5 months	89 (43.0)
6–11 months	62 (30.0)
**Sex**	
Male	114 (55.1)
Female	93 (44.9)
**Place where suspected CRS patient was delivered**	
Private facility	105 (50.7)
Public facility	91 (44.0)
Home	10 (4.8)
Other	1 (0.5)
**Age of mother (yrs)**	
18–25	126 (60.9)
26–30	64 (30.9)
31–35	9 (4.3)
>35	4 (1.9)
Not available	4 (1.9)

Structural heart defects (135; 65.2% of patients with suspected CRS) and eye abnormalities (94; 45.4%) were the most common findings leading to a diagnosis of suspected CRS; 37 (17.9%) children had hearing impairment. Mothers of 41 (19.8%) infants had a history of febrile rash illness during pregnancy (Table 1).

Blood specimens were obtained from 205 (99.0%) of the 207 patients with suspected CRS. IgM antibodies against rubella were identified in 71 (34.6%) patients, and a sustained rise in rubella IgG antibody titers was detected in an additional five (2.4%) patients. Among these 76 patients, 72 (34.8% of 207 with suspected CRS, all of whom met clinical criteria) were classified as laboratory-confirmed CRS, and four were categorized as having congenital rubella infection (positive rubella IgM in the absence of cataracts, congenital glaucoma, pigmentary retinopathy, congenital heart defects, or hearing loss (Box). Eleven (5.3%) patients were considered to have clinically compatible cases; the remaining 120 (58.0%) were excluded as noncases. Most laboratory-confirmed CRS cases were detected at the Chandigarh (33; 45.8%), Jodhpur (17; 23.6%), and Bengaluru (13; 18.1%) sites. The proportion of laboratory-confirmed cases detected among children aged ≤3 months (48 of 115; 41.7%) was significantly higher than that among older children (24 of 92; 26.1%) (p = 0.02).

Among the 72 laboratory-confirmed cases, structural heart defects were present in 60 (83.3%) patients, congenital cataracts in 45 (62.5%), and hearing impairment in 25 (34.7%) (Table 2). Among the 60 laboratory-confirmed CRS patients with structural heart defects, patent ductus arteriosus (PDA) and complex congenital defects with PDA were the most common structural abnormalities detected, accounting for 51 (85%) of all congenital heart defects (Supplementary Table 1, https://stacks.cdc.gov/view/cdc/58461).

**TABLE 2:** Pertinent clinical findings among 207 suspected, 72 laboratory-confirmed, and 120 excluded congenital rubella syndrome (CRS) patients — Congenital Rubella Syndrome Sentinel Surveillance, India, December 2016–July 2017

Clinical finding	All suspected CRS (N = 207), no. (%)	Laboratory-confirmed CRS (N = 72), no. (%)	Excluded noncases (N = 120), no. (%)
**General examination**			
Jaundice	28 (13.5)	5 (6.9)	18 (15.0)
Rash	19 (9.2)	12 (16.7)	4 (3.3)
Lymphadenopathy	7 (3.4)	1 (1.4)	5 (4.2)
Purpura	6 (2.9)	4 (5.6)	0 (—)
**Cardiorespiratory**			
Structural heart defect	135 (65.2)	60 (83.3)	66 (55.0)
Retractions	43 (20.8)	14 (19.4)	23 (19.2)
**Gastrointestinal**			
Hepatomegaly	63 (30.4)	29 (40.3)	28 (23.3)
Splenomegaly	33 (15.9)	17 (23.6)	12 (10.0)
**Central nervous system**			
Microcephaly	91 (44.0)	41 (56.9)	41 (34.2)
Developmental delay	55 (26.6)	19 (26.4)	32 (26.7)
Hypertonia	25 (12.1)	11 (15.3)	10 (8.3)
History of seizures	17 (8.2)	2 (2.8)	10 (8.3)
Hypotonia	14 (6.8)	3 (4.2)	9 (7.5)
Bulging anterior fontanelle	4 (1.9)	0 (—)	2 (1.7)
Meningoencephalitis	4 (1.9)	0 (—)	2 (1.7)
**Ophthalmologic**			
Cataract	78 (37.7)	45 (62.5)	28 (23.3)
Microphthalmos	17 (8.2)	10 (13.9)	6 (5.0)
Pigmentary retinopathy	11 (5.3)	7 (9.7)	3 (2.5)
Congenital glaucoma	7 (3.4)	5 (6.9)	2 (1.7)
Microcornea	8 (3.9)	4 (5.6)	4 (3.3)
**Ear, nose, and throat**			
Hearing impairment	54 (26.1)	25 (34.7)	25 (20.8)
**No. of CRS diagnostic criteria met**			
1	115 (55.5)	21 (29.2)	87 (72.5)
2	61 (29.5)	26 (36.1)	30 (25.0)
≥3	31 (15.0)	25 (34.7)	3 (2.5)

Oropharyngeal swabs were collected from 133 (91.7%) patients aged ≤5 months and shipped to the National Institute of Virology in Pune. Twenty-five (21%) of 119 swabs tested by RT-PCR were positive for rubella. Genotyping of seven representative specimens from northern, western, and southern Indian states revealed all viruses to be the 2B genotype.

During the first 8 months of CRS surveillance in India, adequate data were collected from 207 patients with suspected CRS, and adequate blood specimens (including collection of an additional specimen from infants aged 6–11 months if the first specimen was IgM-negative) were collected from 196 (95%) (Supplementary Table 2, https://stacks.cdc.gov/view/cdc/58462). Although blood specimens were transported to the laboratories within 5 days of collection, only 96 (46.8%) of the 205 results were reported within 4 days of collection. Patients with laboratory-confirmed CRS were not monitored for virus excretion.

## Discussion

This is the first report of long-term CRS surveillance data in India. During the first 8 months of surveillance, 72 laboratory-confirmed cases of CRS were detected at five sentinel sites, which is a substantial increase over the two to three cases that had been passively detected each year in the past by the sentinel sites (Sanjay Verma, Post Graduate Institute of Medical Education and Research, Chandigarh, India, unpublished data, 2018), confirming that CRS is an important public health problem in India. The sentinel surveillance system also generated important epidemiologic data about CRS at these sentinel sites, including information on circulating rubella virus genotypes. An expansion of the surveillance network is planned, and the experience gained during the first phase will help guide this expansion.

Approximately one third of the suspected CRS cases were laboratory-confirmed; this rate of laboratory confirmation was likely related to the use of specific case definitions for suspected CRS, which included infants with confirmed cardiac defects, hearing impairment, or eye abnormalities. This high rate of laboratory confirmation also reflects a substantial risk for rubella in the population.

WHO has proposed eight indicators for assessing the quality of CRS surveillance: 1) reporting rates of suspected CRS cases (as a measure of sensitivity of surveillance); 2) percentage of suspected CRS cases with essential data points recorded (as a measure of adequacy of investigation); 3) proportion of cases that are laboratory-confirmed; 4) proportion of laboratory-confirmed cases with virus detected; 5) proportion of laboratory-confirmed cases monitored for virus excretion; 6) timeliness of detection (after birth); 7) timeliness of specimen transport; and 8) timeliness of laboratory reporting (*9*,*10*). During the first 8 months of CRS surveillance in India, indicator targets were met or surpassed for data adequacy, specimen collection, and timeliness of specimen transport; however, improvement is needed in detection of cases within 3 months of birth, and fewer than half of laboratory results were reported within 4 days. Laboratory diagnosis of CRS in this surveillance was based on serologic tests; oropharyngeal swabs were only collected from children aged ≤5 months for the primary purpose of generating baseline data about circulating genotypes.

The findings in this report are subject to at least two limitations. First, it was not possible to estimate the incidence of CRS. All sentinel sites are tertiary care hospitals and serve large populations, not only from the city where they are located but also from neighboring districts and states. Second, among patients with laboratory-confirmed CRS, heart defects and eye abnormalities were the most common defects, whereas published studies report hearing loss as the most common defect (*2*). This finding points toward a bias in ascertainment of patients with suspected CRS because many of them were recruited from cardiology and ophthalmology clinics and very few from ENT clinics.

The newly initiated sentinel CRS surveillance system is generating quality epidemiologic data about CRS in India. Further expansion of the network and long-term surveillance will be useful to monitor progress made toward control of rubella and CRS in India.

**Summary**

**What is already known about this topic?**

India is committed to eliminating measles and controlling rubella and congenital rubella syndrome (CRS) by 2020. Before 2016, India did not have a systematic CRS surveillance system.

**What is added by this report?**

CRS surveillance in five sentinel sites from 2016 identified 207 suspected CRS cases; 72 (34.8%) were laboratory-confirmed. CRS surveillance met or surpassed indicators for data adequacy, specimen collection, and timeliness of specimen transport. However, timeliness of detection of cases within 3 months of birth and of reporting laboratory results needs improvement.

**What are the implications for public health practice?**

Expansion of the sentinel CRS surveillance network to other states can be guided by experiences during the first 8 months.

## References

[1] Lambert N, Strebel P, Orenstein W, Icenogle J, Poland GA. Rubella. Lancet 2015;385:2297–307. 10.1016/S0140-6736(14)60539-0 25576992 PMC4514442

[2] Banatvala JE, Brown DW. Rubella. Lancet 2004;363:1127–37. 10.1016/S0140-6736(04)15897-2 15064032

[3] World Health Organization. Strategic plan for measles elimination and rubella and congenital rubella syndrome control in the South-East Asia Region. New Delhi, India: World Health Organization Regional Office for South-East Asia; 2015. http://www.searo.who.int/immunization/documents/sear_mr_strategic_plan_2014_2020.pdf

[4] World Health Organization India. India launches one of the world’s largest vaccination campaigns against measles and rubella syndrome with WHO support. New Delhi, India: World Health Organization Regional Office for South-East Asia; 2017. http://www.searo.who.int/india/mediacentre/events/2017/Measles_Rubella/en

[5] Government of India Ministry of Health and Family Welfare. Field guide: measles surveillance & outbreak investigation. New Delhi, India: Government of India Ministry of Health and Family Welfare; 2005. http://www.searo.who.int/india/topics/measles/Measles_surveillance_and_outbreak_investigation_field_guide_2005.pdf

[6] Thapa A, Khanal S, Sharapov U, Progress toward measles elimination—South-East Asia Region, 2003–2013. MMWR Morb Mortal Wkly Rep 2015;64:613–7. 26068565 PMC4584924

[7] Dewan P, Gupta P. Burden of congenital rubella syndrome (CRS) in India: a systematic review. Indian Pediatr 2012;49:377–99. 10.1007/s13312-012-0087-4 22700664

[8] World Health Organization. Manual for the laboratory diagnosis of measles and rubella virus infection. Geneva, Switzerland: World Health Organization; 2007. http://www.who.int/ihr/elibrary/manual_diagn_lab_mea_rub_en.pdf

[9] Castillo-Solórzano C, Reef SE, Morice A, Guidelines for the documentation and verification of measles, rubella, and congenital rubella syndrome elimination in the region of the Americas. J Infect Dis 2011;204(Suppl 2):S683–9. 10.1093/infdis/jir471 21954267

[10] World Health Organization. Guidelines on verification of measles elimination and rubella/congenital rubella syndrome control in the WHO South-East Asia Region. New Delhi, India: World Health Organization Regional Office for South-East Asia; 2016. http://www.searo.who.int/immunization/documents/mr_guidelines.pdf

